# Workplace psychosocial stressors experienced by migrant workers in Australia: A cross-sectional study

**DOI:** 10.1371/journal.pone.0203998

**Published:** 2018-09-20

**Authors:** Alison Daly, Renee N. Carey, Ellie Darcey, HuiJun Chih, Anthony D. LaMontagne, Allison Milner, Alison Reid

**Affiliations:** 1 School of Public Health, Curtin University, Perth, Western Australia, Australia; 2 Centre for Genetic Origins of Health and Disease, Royal Perth Hospital Medical Research Foundation, Perth, Australia; 3 Centre for Population Health Research, Deakin University, Geelong, Victoria, Australia; 4 Centre for Health Equity, School of Population and Global Health, University of Melbourne, Melbourne, Victoria, Australia; National School of Public Health, GREECE

## Abstract

**Objective:**

To explore work-related psychosocial stressors among people of Chinese, Vietnamese and Arabic-speaking backgrounds currently working in Australia.

**Methods:**

In 2015, a telephone survey of 585 Vietnamese, Chinese and Arabic-speaking workers asked about workplace bullying, ethnic discrimination, job complexity, degree of control, security and fairness of payment along with demographic and employment information. Estimates of job-related psychosocial stressors were derived and regression analyses used to identify significant associations.

**Results:**

At least one workplace stressor was reported by 83% of the workers in the study. Education was significantly associated with experiencing any psychosocial stressor and also with the total number of stressors. Workers aged 45 years and older were more likely to be bullied or experience racial discrimination compared with younger workers of any ethnicity. There was a greater likelihood of reporting low control over a job when the interview was conducted in a language other than English and the workers were either Chinese or Arabic. Workers on a fixed-term contract, independent of ethnicity were more likely to report a job with low security. Overall psychosocial job quality decreased with education and was associated with occupation type which interacted with ethnicity and gender.

**Conclusions:**

The results suggest that job-related psychosocial stressors are widespread but not uniform across ethnic groups. Further research into what drives differences in work experience for migrant groups would provide information to guide both employers and migrants in ways to reduce workplace psychosocial stressors.

## Introduction

Unfavourable psychosocial working conditions are established as risk factors for poor physical and mental health [1–4]. A range of workplace psychosocial stressors have been identified, including high work demands, discrimination, bullying, and perceived job insecurity [4, 5]. These workplace psychosocial stressors have been found to vary across a variety of demographic factors, including gender, age, socioeconomic status, and occupational skill level, as well as across differing employment arrangements [6, 7]. There is also some evidence that adverse psychosocial work factors may contribute to existing occupational health disparities among populations already at risk [8, 9], including migrant workers [9, 10]. More recently research has identified workplace psychosocial stress associated with high Body Mass Index and low physical activity [11].

The type or workplace stressor may also be related to specific health effects. Job insecurity has been linked to poor physical health [12, 13], burnout symptoms [14], lower life satisfaction [15], and lower levels of job satisfaction [16]. Long working hours as well as precarious working conditions are associated with depression [17, 18]. Those who report being bullied at work experience greater depressive symptoms [19, 20], absence from work [21] and higher levels of stress and anxiety [22, 23]. Racial discrimination at work has also been linked to mental health effects, with the risk of common mental disorders found to be doubled among those experiencing unfair treatment at work due to their race or ethnicity [24]. The effect of most psychosocial stressors appears to be contemporaneous with the onset of stressors although the impact of job demands appears to increase over time [25].

Workplace psychosocial work factors may be a particular issue for migrant populations who often migrate in order to improve their quality of life and their working conditions. Migrant workers who do not speak the language of the host country, are more likely to work in poorly paid, insecure or precarious jobs in the receiving country [9, 26]. Migrant workers are generally less satisfied with their job and working conditions and work in jobs that are not commensurate with their skills, qualifications and experience [16, 27–29]. This may be compounded by other stressors such as language difficulties and feelings of isolation [30].

Australia is a nation of migrants and one in four of the Australian population is born overseas [31]. Post World War II until the mid-1970s saw more than six million migrants arrive, largely to build Australia’s infrastructure and manufacturing base. From the mid-1990s Australia has focused on skilled migration, and since 2008 two-thirds of all visas issued have been to skilled migrants[32]. In addition, Australia accepts approximately 14,000 Humanitarian migrants each year. In Australia there is limited research investigating workplace psychosocial stressors in migrant workers and none examining these in different groups of migrant workers. This current study is a preliminary investigation workplace psychosocial stressors among Vietnamese, Chinese and Arabic-speaking workers in Australia, who represent distinct migrant groups in the Australian population and make up 20% of the migrant working population. These groups were chosen because our qualitative research had uncovered reports of adverse working conditions among workers from these groups and wanted to explore this more fully in this current study[26]. Specifically, the objectives are to estimate the prevalence of workplace psychosocial stressors for Vietnamese, Chinese and Arabic-speaking workers in Australia and to explore associations between these psychosocial stressors and socio demographics and employment conditions within and between these three migrant worker groups.

## Methods

### Study population

Ethics approval for a cross-sectional telephone survey was obtained from the Human Research Ethics Committee of Curtin University. Oral consent to participate in the study was obtained at the beginning of the interview, which was conducted as a Computer Assisted Telephone Interview. To obtain the sample for this pilot survey, the 2011 Census [33] was used to identify suburbs in Melbourne, Sydney, or Perth with a high concentration of residents of Arabic, Chinese, or Vietnamese background. This list of suburbs was then provided to a commercial survey sampling firm who compiled a list of household telephone numbers publicly available in either telephone books or other publicly available sources and identified surnames reflecting the most common surnames for the target groups. This step was taken because there is no other source of sample by ethnicity presently available in Australia and these groups have recognisably different surnames from the majority of Australian born workers. The sample provided included mobile numbers where available as well as land lines. From this sample frame, a random sample was selected and anyone aged over 18 years old, currently employed, from one of the migrant groups and living in Melbourne, Sydney, or Perth was eligible to participate. As self-report is considered to be the best way to measure race in public health research [34], participants were asked “*Are you Chinese or Vietnamese and over 18 years of age*, *or Arabic-speaking over 18 years of age*?*”* Later in the interview, respondents were also asked where they were born, how long they lived in Australia, what language they spoke at home and how well they spoke English, all of which as used by the Australian Bureau of Statistics to determine ethnicity [35]. Where there was more than one eligible person in the household, the interviewer asked to speak to the one who had the next birthday.

A total of 9,898 households were contacted over the course of the study. There was no response after 10 different call attempts from 4,102 households and 4,348 households had no eligible respondents, leaving 1448 eligible households. Of these, 863 refused to participate and 585 completed the interview (with a quota of 195 for each ethnic group). The adjusted raw response rate was 10.5% (completed interviews ÷ eligible and unknown) and the participation rate was 40.4% (completed interviews ÷ completed + refused). While this response rate is low, in Australia there is no source of specific migrant worker telephone numbers that is accessible to research institutes. The method used in this study is, to date, the only possible which is likely to yield the numbers necessary to provide enough statistical power to investigate the target migrant groups. While this sampling cannot be assumed to be representative of each migrant group, it provides the only information we have on the working conditions of these groups.

### Data collection

The General Ethnic Discrimination Survey 2015, developed by the researchers using questions previously tested for validity and reliability, was conducted as a quantitative, cross sectional computer-assisted telephone interview (CATI) by a survey research company with over fifteen years of experience in CATI. Interviewers were given training in the purpose of the survey and the questionnaire to allow them to respond to any queries from the participants about the purpose of the questions or what would be done with the information. In questions such as country of birth where “please specify” was requested respondents’ answers were recorded verbatim and recoded by an experienced researcher. There were no qualitative questions asked. Following a brief introductory script in English, which also obtained consent to continue, all participants were given the option of completing the interview in English or the language of their choice (Arabic, Vietnamese, Cantonese and Mandarin). When the participant chose to do the interview in a language other than English, a bilingual interviewer conducted the interview. Direct translation of the questionnaire was done at the time of the interview.

Demographic information, including gender, age, country of birth, year of arrival in Australia, language most commonly spoken at home, and highest level of education were collected from all participants. All questions asked in relation to employment were for their current employment or where they held more than one job, the one in which they spent the most time.

Job details collected included the job title of their current occupation, whether the participant worked for an employer or in their own business, the number of other employees they work with, whether or not their employment was full/part-time and whether or not it was casual, a fixed-term contract or permanent.

Occupation information was coded according to the Australian and New Zealand Standard Classification of Occupations (ANZSCO) [36].

#### Description of measures used in estimating psychosocial stressors

All participants answered questions about being bullied, ethnic discrimination and psychosocial job quality indicators. Bullying was defined as repeated negative behaviour directed toward an individual who experiences difficulties defending him/herself [37]. Participants were asked whether they have been subjected to bullying in the workplace in the last six months (*yes/no/refused*), and if so, how often they were exposed to bullying events (*daily*, *at least once per week*, *at least once per month*, *rarely*, *never*), and how long they were exposed to bullying events (*less than 1 month*, *1–6 months*, *7–12 months*, *1–2 years*, *more than 2 years*). A dichotomous variable was created using the response ‘never’ to the frequency bullying question as not bullied and all other response categories for this question as bullied within the last six months. Any don’t know/unsure or refused responses were assigned missing.

Ethnic discrimination was measured using two items from the General Ethnic Discrimination Scale (GEDS). This scale measured unfair treatment at work by employers and by co-workers. Each question was asked three times to: a) ascertain if there was any discrimination, b) the frequency of discrimination in the last year and c) the frequency over a lifetime [38]. The frequency responses used a six-point scale (never, once in a while, sometimes, a lot, most of the time, almost all the time) and were added together to obtain a scale for recent discrimination and discrimination over a lifetime. The reliability estimates for the scales assessing discrimination were in the acceptable internal consistency range (recent discrimination α = 0.74 and lifetime discrimination α = 0.81). A dichotomous variable was created using any reported experience of discrimination in the workplace at any time by anyone as evidence of ethnic discrimination. As the missing values for all other variables were evenly distributed and proportions of missing were not statistically significantly different between groups, any don’t know/unsure or refused responses were assigned missing.

Psychosocial job quality was measured using 10 items assessing job complexity, job control and job security on a seven-point Likert scale ranging from *strongly disagree* to *strongly agree*. Previous research in Australia had identified a three factor structure [39], job complexity (how stressful, complex and difficult the job is, whether or not new skills must be acquired and not being able to use existing skills), job control (being able to determine how and when the work is done and having input into the job) and perceived job security (worried about security of job, think company will be there in five years). The three factors of psychosocial job quality in this study, while being composed of the same structure and items within each, had lower internal consistency estimates than the Butterworth et al (2011) study (job complexity, four items α = 0.56 compared with α = 0.70; job control three items α = 0.67 compared with 0.82; and job security four items, α = 0.56 compared with 0.59). Perceived fairness of pay was used as a separate psychosocial job quality measure and included in the total psychosocial job quality estimate [40].

Two methods of assessing psychosocial job quality were employed. Each of the three factors, complexity, low control and low security, were examined individually using the dichotomised measure for each. Then individual scores from each of the ten questions plus the item about unfair pay were summed to provide an overall measure of psychosocial job quality as a continuous scale with higher scores indicating better total psychosocial job quality. Both methods of measuring psychosocial job quality have been previously validated in Australia [41, 42].

Overall prevalence was defined as the percentage of respondents who reported one or more of the workplace psychosocial stressors (being bullied, racial discrimination, job complexity, job control, job security and unfair pay).

### Statistical analysis

All analyses were conducted using Stata V.14 [43]. Missing data for age (n = 71, 12%) and year of arrival in Australia (n = 15, 3%) were assumed to be random. Imputation of these missing variables was conducted with multiple imputation by chained equations using language spoken at home, location, ethnicity, gender, country of birth, education, industry of employment, type of employment, contract type, company size and occupation [44]. Twenty datasets which contained all demographic and job variables were added and these were averaged with standard errors calculated using Rubin’s adjustment [45].

Univariate descriptive analysis produced estimates with 95% confidence intervals for socio demographic and employment variables and psychosocial stressors. Chi square and ANOVA were used to compare the overall prevalence and prevalence of individual factors by ethnicity. To identify associations with psychosocial stressors, logistic, negative binomial and linear regression analyses were used as appropriate with 95% confidence intervals and the statistical significance level (*p* value). For logistic regression, the odds ratios are provided, for the linear regression, coefficients are provided and for the negative binomial regression, incidence rate ratios are provided. All variables in the univariate analysis were used as covariates in the regression models. Distribution of the psychosocial job quality scale was checked for normality. Post estimation tests were conducted for fit using contrast Hosmer-Lemeshow chi square for logistic models [46]; Bayesian information criteria and contrast for the negative binomial regression model; and normal distribution, missing variables collinearity and contrast for the linear regression model [47, 48]. Deletion of non-significant covariates was used to improve model precision with *p*<0.05 considered statistically significant for all analyses. Interaction terms for ethnicity and gender with all other covariates described in the univariate analysis were explored and reported where significant in both the models and post estimation tests.

## Results

### Demographic characteristics

From the total of 585 respondents (195 from each ethnic group), 63 (10.7%) were born in Australia. Most came from Arabic-speaking countries (n = 39, 63% of those born in Australia). There were no statistically significant differences in the prevalence of psychosocial stressors between the groups born in Australia when compared with those workers who were not born in Australia. Of the Arabic-speaking sample who were born outside Australia almost all were from the Middle Eastern and North African countries (95.5%). For the purpose of this paper, respondents will be referred to as Vietnamese, Chinese or Arabic-speaking workers. Of the 289 interviewed in a language other than English 151 were Vietnamese workers interviewed in Vietnamese (52.3%), 101 were Chinese workers interviewed in either Mandarin or Cantonese Chinese (34.9%) and 37 were from Arabic-speaking countries interviewed in Arabic (12.8%). Vietnamese workers reported estimates that were different from Chinese and Arabic-speaking workers in both demographic and work characteristics. These included being older, have less schooling and having spent longer in Australia. Chinese workers were more likely to have completed tertiary education compared with their Vietnamese and Arabic counterparts. Arabic-speaking workers were more likely to be interviewed in English compared with Vietnamese and Chinese workers. The demographic and employment characteristics of the sample are shown in Table 1 with 95% confidence intervals and significance estimates.

**Table 1:** Prevalence of demographic and employment characteristics among migrant workers in Australia, General Ethnic Discrimination Survey 2015.

	Vietnamese	Chinese	Arabic-speaking	
**Gender (n = 585)**	**% [ci]**	**% [ci]**	**% [ci]**	***p***
Male	46.2 [39.2,53.2]	42.6 [35.8,49.6]	**59.0 [51.9,65.7]**	
Female	53.8 [46.8,60.8]	57.4 [50.4,64.2]	41.0 [34.3,48.1]	0.003
**Age Group**^**a**^ **(n = 583)**				
18–34	**14.6 [9.2,20.0]**	34.6 [27.4,41.8]	35.0 [28.0,41.9]	
35–44	21.5 [15.3,27.7]	19.5 [13.7,25.2]	21.3 [15.2,27.3]	
45–54	31.3 [24.3,38.3]	24.8 [18.1,31.5]	29.0 [22.4,35.6]	
55–65	**32.4 [24.9,39.8]**	21.0 [15.0,27.0]	14.6 [9.3,19.8]	<0.0001
**Education level (n = 578)**				
Less than year 12	23.6 [18.1,30.1]	**2.6 [1.1,6.1]**	**11.9 [8,17.2]**	
Year 12	29.8 [23.8,36.7]	18.7 [13.7,24.8]	19.1 [14.1,25.2]	
Trade/Technical	9.9 [6.4,15.1]	13.0 [8.9,18.5]	20.1 [15.0,26.4]	
Tertiary	36.6 [30.1,43.7]	**65.8 [58.8,72.2]**	49.0 [42.0,56.0]	<0.0001
**Years in Australia**^**a**^ **(n = 584)**				
Born in Australia	6.7 [3.9,11.2]	5.6 [3.1,9.9]	**20.0 [14.9,26.2]**	
Up to 12 years	**10.9 [6.3,15.4]**	32.2 [25.5,38.8]	25.3 [19.1,31.4]	
Between 12 and 22 years	20.4 [14.3,26.4]	30.6 [24.1,37.2]	20.4 [14.7,26.2]	
Between 23 and 30 years	29.1 [22.5,35.7]	23.1 [17.2,29.1]	13.4 [8.6,18.2]	
Over 30 years	**32.7 [26.0,39.4]**	8.2 [4.3,12.1]	20.7 [15.0,26.4]	<0.0001
**Language spoken at home (n = 585)**				
Language other than English	80.0 [73.8,85.1]	41.0 [34.3,48.1]	42.1 [35.3,49.1]	
English	**20.0 [14.9,26.2]**	59.0 [51.9,65.7]	57.9 [50.9,64.7]	<0.0001
**Language of interview (n = 585)**				
Language other than English	**77.4 [71,82.8]**	51.8 [44.8,58.8]	**19.0 [14.0,25.1]**	
English	22.6 [17.2,29]	48.2 [41.2,55.2]	81.0 [74.9,86.0]	<0.0001
**Work for self or employer (n = 585)**				
Work for an employer	82.1 [76,86.8]	85.6 [80,89.9]	80.5 [74.3,85.5]	
Self-employed	17.9 [13.2,24]	14.4 [10.1,20]	19.5 [14.5,25.7]	ns0.389
**Number of other employees (n = 579)**				
No other employees	7.8 [4.8,12.6]	8.2 [5.1,13.1]	10.9 [7.2,16.1]	
Less than 5	21.4 [16.1,27.7]	**10.3** [6.7,15.5]	12.4 [8.5,17.9]	
5 to 20	33.3 [27.0,40.3]	26.8 [21,33.5]	25.9 [20.2,32.6]	
20 to 200	25.5 [19.8,32.2]	30.4 [24.3,37.3]	28.0 [22.1,34.8]	
200+	**12.0** [8.1,17.4]	24.2 [18.7,30.8]	22.8 [17.4,29.3]	0.005
**Occupation (n = 581)**				
Managers	6.2 [3.5,10.5]	5.7 [3.2,10.1]	11.9 [8.0,17.2]	
Professionals	**14.4 [10.1,20.0]**	34.9 [28.5,41.9]	27.3 [21.5,34]	
Technicians/trades workers	14.9 [10.5,20.6]	11.5 [7.6,16.8]	16.0 [11.4,21.9]	
Community/personal service workers	15.9 [11.4,21.8]	17.2 [12.5,23.2]	13.4 [9.3,19.0]	
Clerical/administrative workers	6.7 [3.9,11.2]	7.3 [4.4,12.0]	5.7 [3.2,10.0]	
Sales workers	10.3 [6.7,15.4]	6.8 [4.0,11.3]	13.4 [9.3,19.0]	
Machinery operators & drivers	8.2 [5.1,13.0]	4.7 [2.4,8.8]	6.7 [3.9,11.2]	
Labourers	**23.6 [18.1,30.1]**	12 [8.1,17.4]	5.7 [3.2,10.0]	<0.0001
**Job type (n = 578)**				
Full-time	69.6 [62.7,75.8]	65.6 [58.7,72]	62.0 [54.9,68.6]	
Part-time	30.4 [24.2,37.3]	34.4 [28,41.3]	38.0 [31.4,45.1]	ns0.288
**Type of employment contract (n = 577)**				
Casual	19.3 [14.3,25.5]	16.0 [11.4,21.9]	23.0 [17.6,29.6]	
Fixed Term Contract	**0.50** [0.1,3.6]	17.0 [12.3,23.0]	8.9 [5.6,13.9]	
Permanent	62.0 [54.9,68.6]	52.6 [45.5,59.5]	48.2 [41.1,55.3]	
Self-employed	18.2 [13.4,24.4]	14.4 [10.1,20.1]	19.9 [14.8,26.2]	<0.0001
**Hours worked weekly (n = 567)**				
Up to 20 hours	21.7 [16.3,28.3]	18.7 [13.7,24.8]	24.2 [18.6,30.8]	
21 to 40 hours	66.8 [59.7,73.3]	63.7 [56.7,70.2]	57.9 [50.7,64.7]	
Over 40 hours	11.4 [7.5,16.9]	17.6 [12.8,23.7]	17.9 [13.1,24.0]	0.747

Estimates in bold show groups that differ from one or more of the other migrant worker groups.

### Prevalence of workplace psychosocial stressors

A total of 432 (82.9% [79.4, 85.9]) participants reported experiencing at least one of the workplace psychosocial stressors. Vietnamese workers were least likely to report any workplace psychosocial stressors (81.1% [74.7, 86.2] compared with Chinese workers (86.7% [81.1, 90.8]) and Arabic-speaking workers (86.5% [80.9, 90.7]), although the difference was not statistically significant (χ^2^ = 2.88, *p* = 0.237). Being bullied in the workplace within the last six months showed no significant differences between the migrant worker groups. The frequency of experiencing discrimination was significantly higher for Arabic-speaking workers compared with Vietnamese or Chinese workers. Of those experiencing any psychosocial job quality indicator, 44.8% [40.2, 49.6] experienced one, 43.7% [39.1, 48.4] experienced two and 11.5% [8.8, 14.9] experienced three. There was no significant difference by ethnicity for number of psychosocial job quality factors reported (F = 2.71, *p* = 0.07).

The most frequently reported combination (regardless of ethnicity) was low job security in combination with ethnic discrimination, followed by low control combined with ethnic discrimination. Differences by gender were also found, with males more likely to report a combination of ethnic discrimination and both high complexity and low security compared with females.

### Being bullied and ethnic discrimination

Of all the workplace stressors, being bullied was reported by the smallest percentage of workers within each ethnicity while ethnic discrimination was reported by the highest percentage within each ethnicity.

Using logistic regression, ethnicity, age, and main language spoken at home were significantly associated with being bullied. Specifically, Chinese workers were more likely to report being bullied (OR = 1.98 [1.02, 3.86]) than either Vietnamese (reference group) or Arabic-speaking workers (OR = 1.63 [0.82, 3.24], *p* = 0.045). Workers aged 45 years and over were more likely to report being bullied (OR = 1.84 [1.06, 3.19], *p* = 0.031) compared with workers aged 18 to 44 years. Speaking English as the main language at home compared with speaking another language at home decreased the likelihood of being bullied (OR = 0.5 [0.28, 0.89], *p* = 0.018).

Ethnic discrimination was significantly associated with age and education level. Workers aged 45 years and over were more likely to report ethnic discrimination (OR = 1.47 [1.03, 2.08], *p* = 0.032) compared with workers aged 18–44 years. There was also an increased likelihood of ethnic discrimination as education level increased from less than 12 years of schooling to having a tertiary education (OR = 1.35 [1.16, 1.57], *p* = 0.000).

The models for being bullied and ethnic discrimination, while demonstrating a goodness of fit statistically, described little of the variance.

### Variables associated with psychosocial job quality

Associations with psychosocial quality of life factors and socio demographic and employment conditions are show on Table 2. Associations previously found with psychosocial quality of life factors such as level of education, age, occupation and employment conditions were confirmed in this model. In addition, the longer migrants lived in Australia, the more likely they were to have jobs with high demands.

**Table 2:** Associations between socio demographics and employment and the three factors of psychosocial job quality among ethnic minority workers, with interaction terms.

	High complexity*		Low control*		Low security*	
**Covariates**	**OR [95% CI]**	***p***	**OR [95% CI]**	***p***	**OR [95% CI]**	***p***
**Vietnamese**	**Reference**		**Reference**		**Reference**	
Chinese	0.35[0.21,0.59]	0.000	0.64 [0.3,1.39]	0.262	0.10 [0.02,0.56]	0.009
Arabic-speaking	0.77[0.47,1.25]	0.291	0.38 [0.18,0.78]	0.009	1.06 [0.25,4.44]	0.941
**18–44 years**			**Reference**			
45 years & over			2.07 [1.32,3.25]	0.001		
**Only school education**	**Reference**					
Some qualification after school	2.12 [1.32,3.40]	0.002				
**Born in Australia**	**Reference**					
Up to 12 years	1.77 [0.82,3.86]	0.149				
13 to 22 years	1.72 [0.80,3.70]	0.167				
23 to 30 years	3.05 [1.42,6.57]	0.004				
Over 30 years	2.32 [1.08,5.00]	0.032				
**Interviewed in English**			**Reference**			
Interviewed in other language			0.04 [0.02,0.12]	0.000		
Interviewed in other language*Vietnamese			**Reference**			
Interviewed in other language *Chinese			26.03 [8.08,83.86]	0.000		
Interviewed in other language *Arabic-speaking			17.2 [4.27,69.29]	0.000		
**Other language mainly spoken at home**					**Reference**	
English					0.56 [0.37,0.86]	0.008
	High complexity**		Low control**		Low security**	
**Covariates**	**OR [95% CI]**	***p***	**OR [95% CI]**	***p***	**OR [95% CI]**	***p***
**Managers**	**Reference**					
Professionals	1.07 [0.52,2.19]	0.850				
Technicians & trades workers	0.58 [0.27,1.29]	0.183				
Community & personal service workers	0.39 [0.17,0.89]	0.026				
Clerical & administrative workers	0.65 [0.25,1.68]	0.372				
Sales workers	0.30 [0.12,0.78]	0.013				
Machinery operators & drivers	0.30 [0.10,0.86]	0.025				
Labourers	0.41 [0.17,0.98]	0.044				
**Works for others**	**Reference**					
Self-employed	1.92 [1.17,3.14]	0.009				
**Self-employment**			**Reference**		**Reference**	
Casual contract			4.99 [2.21,11.3]	0.000	1.35 [0.66,2.78]	0.415
Fixed term contract			4.2 [1.67,10.56]	0.002	2.89 [1.32,6.36]	0.008
Permanent			4.07 [1.95,8.46]	0.000	0.88 [0.50,1.55]	0.662
**Weekly hours worked & ethnicity**					**Reference**	
Weekly hours worked*Vietnamese					0.98 [0.95,1.02]	0.273
Weekly hours worked *Chinese					1.04 [1.01,1.08]	0.007
Weekly hours worked *Arabic-speaking					1.00 [0.98,1.02]	0.864

a Multiple imputation regression analysis was used for age and years in Australia

* Interaction term

** High complexity logistic regression n = 572, F = 401, p<0.0001; Low control logistic regression n = 574, F = 7.31 p<0.0001; Low security logistic regression n = 517, F-4.05 p<0.0001.

However, all three factors also varied with ethnicity. Compared with Vietnamese workers, Chinese workers were less likely to report high complexity and low security while Arabic-speaking workers were less likely to report low control. Chinese and Arabic speaking workers who were interviewed in a language other than English were very much more likely to report low job control compared with Vietnamese workers interviewed in a language other than English. Chinese workers also reported an increased likelihood of low job security compared with their counterparts who worked the same hours.

### Factors associated with overall psychosocial job quality

The linear regression model showing associations between overall psychosocial job quality (the summed scale of high demand, low control and low security ratings) and socio demographic and employment conditions is presented on Table 3. Poorer psychosocial job quality was associated with level of gender, education, ethnicity and occupation (unless you were male in certain occupations). The linear regression identified three statistically significant interaction terms with ethnicity: language of interview, occupation and employment contract type. Compared with Vietnamese workers interviewed in a language other than English, Chinese and Arabic-speaking workers had lower psychosocial job quality. Compared with workers in managerial occupations (the reference group), all other occupations were associated with lower psychosocial job quality. However ethnicity mitigated the effect of occupation on psychosocial job quality for Chinese workers in professional, technical, community service administrative or labourer occupations and Arabic speaking workers in labour occupations when compared with Vietnamese workers in the same occupations. Having a fixed-term contract was associated with the highest decrease in psychosocial job quality but this was mitigated if country of birth was either China or Arabic-speaking country compared with their Vietnamese counterparts.

**Table 3:** Psychosocial job quality (the summed scale of high demand, low control and low security ratings) with socio demographic and employment associations among migrant workers with significant interactions.

	Coefficient [95%]	*p*
**Vietnamese**	**Reference**	
Chinese	-8.30 [-15.52,-1.09]	0.024
Arabic-speaking	-4.41 [-11.87,3.04]	0.246
**Female**	**Reference**	
Male	-8.51 [-13.49,-3.52]	0.001
**Only school education**	**Reference**	
Trade/Dip/University	-1.99 [-3.55,-0.44]	0.012
**Interviewed in English**	**Reference**	0.000
Interviewed in other language	6.27 [3.24,9.30]	0.000
Vietnamese interviewed in other language	**Reference**	
Chinese & interviewed in other language	-5.78 [-9.33,-2.23]	0.001
Arabic-speaking & interviewed in other language	-4.28 [-7.53,-1.03]	0.010
**Language other than English spoken at home**	**Reference**	
English spoken	2.21 [0.40,4.01]	0.017
**Managers**	**Reference**	
Professionals	-12.83 [-18.26,-7.41]	0.000
Technicians & trades workers	-12.71 [-20.78,-4.65]	0.002
Community & personal service workers	-13.84 [-20.02,-7.67]	0.000
Clerical & administrative workers	-11.55 [-18.84,-4.26]	0.002
Sales workers	-8.68 [-14.75,-2.60]	0.005
Machinery operators & drivers	-6.39 [-18.55,5.77]	0.303
Labourers	-15.43 [-21.26,-9.6]	0.000
**Compared with females of same occupation**	**Reference**	
Male Professionals	5.86 [0.19,11.53]	0.043
Male Technicians & trades workers	8.29 [1.78,14.79]	0.013
Male Community & personal service workers	11.98 [5.21,18.76]	0.001
Male Labourers	8.90 [3.02,14.79]	0.003
**Compared with Vietnamese of same occupation**	**Reference**	
Chinese Professionals	10.32 [2.58,18.05]	0.009
Chinese Technicians & trades workers	14.47 [6.73,22.21]	0.000
Chinese Community & personal service workers	7.69 [0.39,14.99]	0.039
Chinese Clerical & administrative workers	11.05 [2.34,19.75]	0.013
Chinese Labourers	11.14 [2.10,20.17]	0.016
Arabic-speaking Labourers	9.48 [1.82,17.14]	0.015
**Self-employed**	**Reference**	
Casual	-4.65 [-9.02,-0.27]	0.037
Fixed Term Contract	-30.56 [-36.27,-24.86]	0.000
Permanent	-0.77 [-4.72,3.18]	0.703
**Compared with Vietnamese of same contract type**	**Reference**	
Chinese Casual	7.03 [0.99,13.07]	0.023
Chinese Fixed Term Contract	27.61 [20.87,34.35]	0.000
Arabic-speaking Fixed Term Contract	28.33 [20.6,36.05]	0.000
**Constant**	**49.9 [43.93,55.88]**	**0.000**

### Variables associated with total number of workplace psychosocial stressors

Table 4 provides the incidence rate ratios for variables associated with the number of workplace psychosocial stressors, which ranged from zero to six.

**Table 4:** Number of psychosocial stressors# 0 to 6 (bullied, ethnic discrimination, complexity, control, security and unfair pay), among migrant workers with interactions.

Covariates	IRR [95% CI]	*p*
**Vietnamese**	**Reference**	
Chinese	0.83 [0.23,2.93]	0.768
Arabic-speaking	2.92 [1.55,5.49]	0.001
**Aged 18–44 years**	**Reference**	
Aged 45 years and over	1.19 [1.04,1.38]	0.015
**Language other than English spoken at home**	**Reference**	
English	0.87 [0.76,1.00]	0.050
**Less than year 12 schooling**	**Reference**	
Year 12	1.83 [1.14,2.92]	0.012
Diploma/Certificate/Trade	1.92 [1.11,3.32]	0.020
Tertiary	2.23 [1.42,3.51]	0.001
**Vietnamese of comparable education**	**Reference**	
Chinese*Year 12	1.60 [0.46,5.59]	0.461
Chinese* Diploma/Certificate/Trade	1.44 [0.39,5.28]	0.583
Chinese*Tertiary	1.30 [0.38,4.45]	0.672
Arabic-speaking*Year 12	0.40 [0.22,0.76]	0.005
Arabic-speaking * Diploma/Certificate/Trade	0.49 [0.25,0.96]	0.039
Arabic-speaking *Tertiary	0.46 [0.26,0.81]	0.008
**Self employed**	**Reference**	
Casual	1.44 [0.94,2.20]	0.097
Fixed term	4.51 [1.71,11.86]	0.002
Permanent	1.17 [0.81,1.69]	0.389
**Vietnamese of same contract type**	**Reference**	
Chinese*Casual	0.76 [0.42,1.37]	0.361
Chinese*Fixed Term Contract	0.26 [0.09,0.73]	0.011
Chinese*Permanent	0.96 [0.58,1.56]	0.857
Arabic-speaking *Casual	0.68 [0.40,1.17]	0.162
Arabic-speaking *Fixed Term Contract	0.22 [0.08,0.63]	0.005
Arabic-speaking *Permanent	0.88 [0.55,1.39]	0.576

#Negative binomial regression n = 512, F = 1.85 p = 0.009.

* Interaction.

Arabic-speaking workers had almost three times the risk of experiencing more workplace psychosocial stressors compared with their Vietnamese and Chinese counterparts. There was an increased risk of experiencing more workplace psychosocial stressors as the level of education increased and older workers also had an increased risk. Ethnicity interacted with education and contract type. Compared with Vietnamese workers at the same education level, Chinese workers had a higher risk of experiencing more workplace psychosocial stressors although the increased risk was not statistically significant. Arabic-speaking workers had a significantly decreased risk of experiencing workplace psychosocial stressors compared with their Vietnamese counterparts. While working under any type of contract was associated with a higher risk of experiencing more workplace psychosocial stressors when compared to self-employed workers, compared with their Vietnamese counterparts, Chinese or Arabic-speaking workers in a fixed-term contract had a significantly reduced risk of experiencing workplace psychosocial stressors.

## Discussion

The present study investigated work-related psychosocial stressors for Vietnamese, Chinese and Arabic-speaking workers in Australia. 82.9% of Arabic-speaking, Chinese, and Vietnamese workers surveyed were exposed to at least one workplace psychosocial stressor. The associations with ethnicity varied by type of psychosocial stressor and, differences existed between male and female workers within ethnic groups.

Approximately 12% of Vietnamese, Chinese and Arabic-speaking workers reported being bullied at work in the last six months with no significant difference between the ethnic groups. This prevalence is lower than that found for ethnic respondents in Wales (35%) compared with the 9% reported by “white” respondents [49] but higher than a previously reported 6% estimate for the general Australian working population [50] as well as the United Kingdom (UK) (10.6%) and Danish (8.3%) working populations [51]. Our finding is consistent with the self-reported studies when ‘bullying’ was operationally defined for the respondent (as in the present study) compared with studies that did not include a definition [52]. The lower prevalence reported in studies when bullying is defined may be due the wording of the definition which is usually quite specific and contains power and time dimensions that must be met [37, 53]. In contrast, although there is a close relationship between being bullied and experiencing ethnic discrimination, ethnic discrimination does not require either a power or time dimension [54]. This may account for the large discrepancy between the prevalence of ethnic discrimination (55%) compared with being bullied (12%) found in the current study.

There was no difference in the percentage who reported discrimination between ethnic groups. These results are consistent with previous research in Australia that found that between 40% and 52% of refugee workers reported experiencing work-related discrimination [55]. Similarly, between 2% and 32% of Australian migrants reported being treated unfairly at work in the last year, with levels highest for those born in China and Hong Kong (16%), India (17%) and South Sudan (32%) [56]. These levels are however appreciably higher than those previously reported among white workers, with a study in the United States finding 11% of white workers reported having ever experienced ethnic discrimination at work [57] and a UK study finding that 1.5% of white British workers reported unfair treatment at work as a result of their ethnicity [24]. In Australia, to date there are no reported estimates of ethnic discrimination for white Australian workers but an exploratory study with university students in Australia found that white students reported the lowest rates of discrimination compared with all other groups including ethnic groups, women and ‘international’ students[58]. Work with white Australian workers and ethnic discrimination among other psychosocial stressors is currently in preparation.

Psychosocial job quality varied by its components and also by ethnicity interacting with other variables such as gender and age indicating that this is a complex area. For the factor job complexity, education level above schooling and living in Australia more than 22 years were associated with higher reported complexity. Working as a professional of any kind was also associated with high complexity which would be expected as would the higher education levels. It may be that living longer in Australia provides an opportunity for of either getting more education and/or being promoted but that would need to be investigated. Other work has shown that between 36% and 49% of newly arrived migrants to Australia reported using their skills in their job sometimes, rarely or never [27]; and using skills at work has been significantly associated with job satisfaction [16]. In this study, job complexity was associated with having an education level higher than schooling which may be consistent with skill level. The assumption here is that complexity is a negative component of psychosocial job quality and it may be for persons who are being asked to perform tasks with a complexity that is not commensurate with their skill level (and/or ability to attain the necessary skill level). A study based in Scotland found that highly skilled migrant workers get segmented into low skilled jobs with bullying and racial discrimination [59]. However, for migrant workers who are in jobs commensurate with their skill level, complexity may not be perceived as a negative component of job quality. The inter-relationship between job complexity, education level and occupation in migrant workers needs to be further investigated to understand the alignment of complexity as a workplace psychosocial stressor.

Low job control was reported more often by Chinese or Arabic-speaking workers who had elected to be interviewed in a language other than English compared with Vietnamese workers. Workers over the age of 55 years were more likely to report low control over their job when interviewed in a language other than English although there was no interaction between education levels and being interviewed in a language other than English. If control over a job requires a level of understanding in terms of requirements for job tasks, our results suggest that not understanding English well enough to do an interview could possibly also be related to finding instructions difficult to understand in a job which could be further exacerbated by being older. The complicated relationships shown in the model for reporting low job control needs further investigation as this particular workplace psychosocial stressor has been associated with an increased risk of suicide [60].

We found that 28% of Vietnamese, Chinese and Arabic-speaking workers experience low job security. This is higher than previously found in the general Australian population in 2012 [61] which reported low job security for 22% males compared with 30.6% in this study and 16% females compared with 25.7% in this study. However, these estimates are similar to that reported elsewhere, with migrant workers generally reported to be less satisfied with their job security than native born-workers [16], a disparity that may persist up to 21 years after arrival in Australia [62]. Our results did not find a similar association with time in Australia not significantly related to low job security in this study. The reason for this finding needs further investigation but may be due to the specific migrant workers that were the subject of this study. Low job security was also more likely to be reported by workers on a fixed-term contract compared with any other contract type, while those who spoke English as their main language at home were less likely to report low job security. While it might be expected that speaking English at home would be associated with other variables such as education, gender and ethnicity, our study did not find any significant interactions with these variables. Further research is needed to explore the significance of speaking English at home and workplace psychosocial stressors on job security.

Higher education levels were associated with ethnic discrimination, job complexity and lower psychosocial job quality, as well as reporting a higher number of workplace psychosocial stressors. These results which were independent of country of birth require further investigation.

Age was also a major predictor of workplace psychosocial stressors, with those aged 45 and over more likely to experience being bullied, ethnic discrimination, and low job control, as well as reporting a higher number of stressors, independent of ethnicity and place of birth. As this was not related to time in Australia or education level, it suggests that other factors related to age may be important. A longitudinal analysis of psychosocial working conditions showed similar results with older people reporting low job control and job security which were not attenuated over time while similar conditions in younger people showed some attenuation. [7]

There are some limitations of this study which may have influenced the results. The study was conducted by telephone and the lack of an established sampling frame from which to randomly sample as well as the low response rate may have been affected the representativeness of the sample, a challenge common in ethnic minority research [63]. While it can be argued that a face-to-face survey would include those with no telephones, thus including the most disadvantaged workers, in Australia there is no publicly available source of contact information associated with migrant status.

In Australia according to the most recent published census of 2016, Vietnamese workers made up about 1.3% of the working population, Chinese workers about 3.7% and Arabic-speaking workers about 1.9% [64]. Although our sample was generally representative of the working population of these migrant workers in Australia compared with the labour force statistics from the 2011 Census [65], workers who were younger, male workers, and workers with tertiary education are under-represented while workers with lower levels of education and older workers were over-represented.

The sampling method used in this study was based on evidence of some clustering in areas of residence among migrants in Australia [66]. Therefore the Census was used to identify areas with a high concentration of the study’s ethnic minority populations in order to guide our sampling. This was further supplemented by seeking the most common surnames associated with these migrant groups. While this strategy probably increased the likelihood of reaching a worker from the target ethnic background, it may also have introduced a bias towards underestimation of prevalence of some factors, particularly discrimination due to ethnic density [67, 68].

As this study was an exploratory study, the relatively small sample size meant that some results need to be interpreted with caution given the wide confidence intervals. The small sample size also influenced the analyses of interactions between the variables which should be regarded as exploratory and suggestive of relationships to guide further investigation. For the same reason, exploratory investigation of each ethnic group separately was not conducted but would be very useful and should include collecting information on citizenship as well as years in Australia as this may affect results. Finally, the use of direct translation of the questionnaire during the interview, due to costs and time, may have influenced the results observed in this study [69] particularly as it was done more often for Vietnamese workers, than the other groups.

Strengths of this study include the sampling of workers from the community, rather than sampling workers in particular industries or occupations. This provided an overview of the prevalence of workplace psychosocial stressors across a wider segment of workers than would otherwise have been possible. Offering multiple languages for completion of the interview enabled us to include those workers who may have been excluded from other research as a result of their English language ability. This is particularly important as almost half of all participants chose to complete the interview in a language other than English indicating that even those who perceived that they spoke English well (28%) or very well (42.9%) are more comfortable to speak on the telephone in their preferred language.

## Conclusions

Where you were born does make a difference to work experience in Australia. Since the mid-1990s, Australia’s migration focus has been on attracting skilled workers and today they come from more than 180 countries. While improving quality of life, is one of the main drivers for migration, the process of migration is expensive and as a consequence many migrants take ‘any’ job in order to offset those costs. We have found that approximately four fifths of the migrant workers in this study reported experiencing at least one workplace psychosocial stressor. More than half had been exposed to ethnic discrimination at work in their lifetime, while 28% experienced low job security, a number higher than previously found among the general Australian working population. Vietnamese workers were least likely to report any workplace psychosocial stressors. There were interactions between ethnicity and gender which further underline the need to avoid assuming that migrant workers are uniform and experience the same workplace psychosocial stressors.

Our study shows that work-related psychosocial stressors are widespread, but not uniform, among migrant workers in Australia. Further research into what drives differences in work experience for migrant groups would provide information to guide both employers and migrants in ways to reduce workplace psychosocial stressors. Occupational health and safety agencies could work more closely with employer and employee organisations to encourage and enforce psychosocially healthy workplaces that welcome diversity in order to maintain a healthy workforce for all.
